# Algometry to measure pain threshold in the horse’s back – An in vivo and in vitro study

**DOI:** 10.1186/s12917-017-1002-y

**Published:** 2017-03-29

**Authors:** Una Pongratz, Theresia Licka

**Affiliations:** 1 Equine Clinic, University of Veterinary Medicine Vienna (Vetmeduni Vienna), Veterinaerplatz 1, 1210 Vienna, Austria; 2 Department of Veterinary Clinical Science, Royal (Dick) School of Veterinary Studies, University of Edinburgh, Easter Bush, Edinburgh, EH25 9RG UK

**Keywords:** Thoracolumbar pain, Horse, Algometry

## Abstract

**Background:**

The aim of this study was to provide information on algometric transmission of pressure through the dorsal thoracolumbar tissues of the equine back. Using a commercially available algometer, measurements were carried out with six different tips (hemispheric and cylindrical surfaces, contact areas 0.5 cm^2^, 1 cm^2^, and 2 cm^2^). In nine live horses the threshold of pressure that lead to any reaction was documented. In postmortem specimens of five euthanized horses the transmission of algometer pressure onto a pressure sensor placed underneath the dorsal thoracolumbar tissues at the level of the ribs or the transverse lumbar processes respectively was measured.

**Results:**

Algometer tips with a contact area of 1 cm^2^ led to widely similar results irrespective of the surface shape; these measurements also had the lowest variance. Contact areas of 0.5 cm^2^ resulted in a lower pressure threshold, and those of 2 cm^2^ resulted in a higher pressure threshold. The hemispheric shape of the contact area resulted in a higher pressure threshold, than the cylindrical contact area.

Compared to the thoracic region, a significantly higher pressure threshold was found in the lumbar region in the live horses. This result corresponds to the increased tissue thickness in the lumbar region compared to the thoracic region, also documented as less pressure transmission in the lumbar region on the in vitro specimens.

**Conclusions:**

Algometry is an easily practicable and well tolerated method to quantify pain but it is important to consider the many factors influencing the results obtained.

## Background

Musculoskeletal disorders of the horse are one of the most common reasons for veterinary intervention, and associated clinical, economic, and ethical aspects are increasingly important [1, 2]. The diagnosis of pain in the thoracolumbar area can be challenging due to the variety of possible clinical signs and the lack of objective parameters [3–8].

Even though a large number of diagnostic aids are available for the investigation of thoracolumbar pain, palpation remains one of the most important methods; this is even described as the most valuable part of the clinical assessment [7]. A standardized manual and digital palpation is recommended [7, 9], however this is limited by a large degree of subjectivity [9]. Algometry has been shown to be an objective method to quantify pressure sensitivity or pain on pressure; it is easy to use and it has been successful in a number of studies in veterinary and human medicine [3–5, 9–11]. The pressure exerted via the algometer at which a first reaction of the subject of the examination is noted is known as the mechanical nociceptive threshold. This pressure is then documented as N (Newton) or kg/cm^2^, and – obviously larger values indicate a higher threshold than smaller values [2]. Even though algometry is a quantitative measure for pain thresholds, it is also influenced by factors of the examiner and the subject examined [12]. Some degree of variation between examiners is to be expected [3], and the consistency of results is increased notably if a single examiner is carrying out all measurements [13]. In veterinary medicine the nature of the animals investigated is also an important factor. While the short term repeatability of algometric examinations is good overall, even these can vary within an individual [14]. Within an individual, an increase of the nocicpetive threshold has been noted from cranial to caudal along the back [4, 5]. Between individuals, factors such as sex, age and breed have been noted to influence algometry results, with a higher threshold described in young, heavy, non-Thoroughbred horses and geldings [4, 5].

A variety of algometer tips have been used in human and veterinary medicine. In human medicine algometer tips above the size of 1.6 mm are thought to measure the summation of sensation of deeper tissues [14–17], while algometer tips 0.2 mm diameter are thought to measure intraepidermal nerve endings [17], while larger algometer tips with surfaces of up to 2.1 cm2 are used to imitate the area of two finger tips [18]. The majority of studies in human and veterinary medicine are using tip sizes of 0.5 cm2 and 1 cm2 [4, 5, 11], but they have not yet been compared in the back of the horse.

The aim of the present study was to document the influence of tissue thickness on the penetration of algometric pressure and to compare these results using hemispherical and cylindrical algometer tips of different diameters.

## Methods

### Animals

Nine horses (seven geldings, two mares, 9–22 years, Standardbred Trotters and Warmblood horses) without a history of muscular problems, thoracolumbar pain, surgery, wounds or trauma of the back were used for this study. None of the horses was used for ridden exercise in the year prior to the data collection.

For the post mortem part of the study, six trunks of horses (three mares, three geldings, Warmbloods, Standarbred Trotters, Coldblood, 3–10 years) were obtained; in these cases euthanasia was carried out for reasons other than musculoskeletal problems; also these horses had no known history of, thoracolumbar pain, surgery, wounds or trauma of the back.

### Equipment

**Fig. 1:**
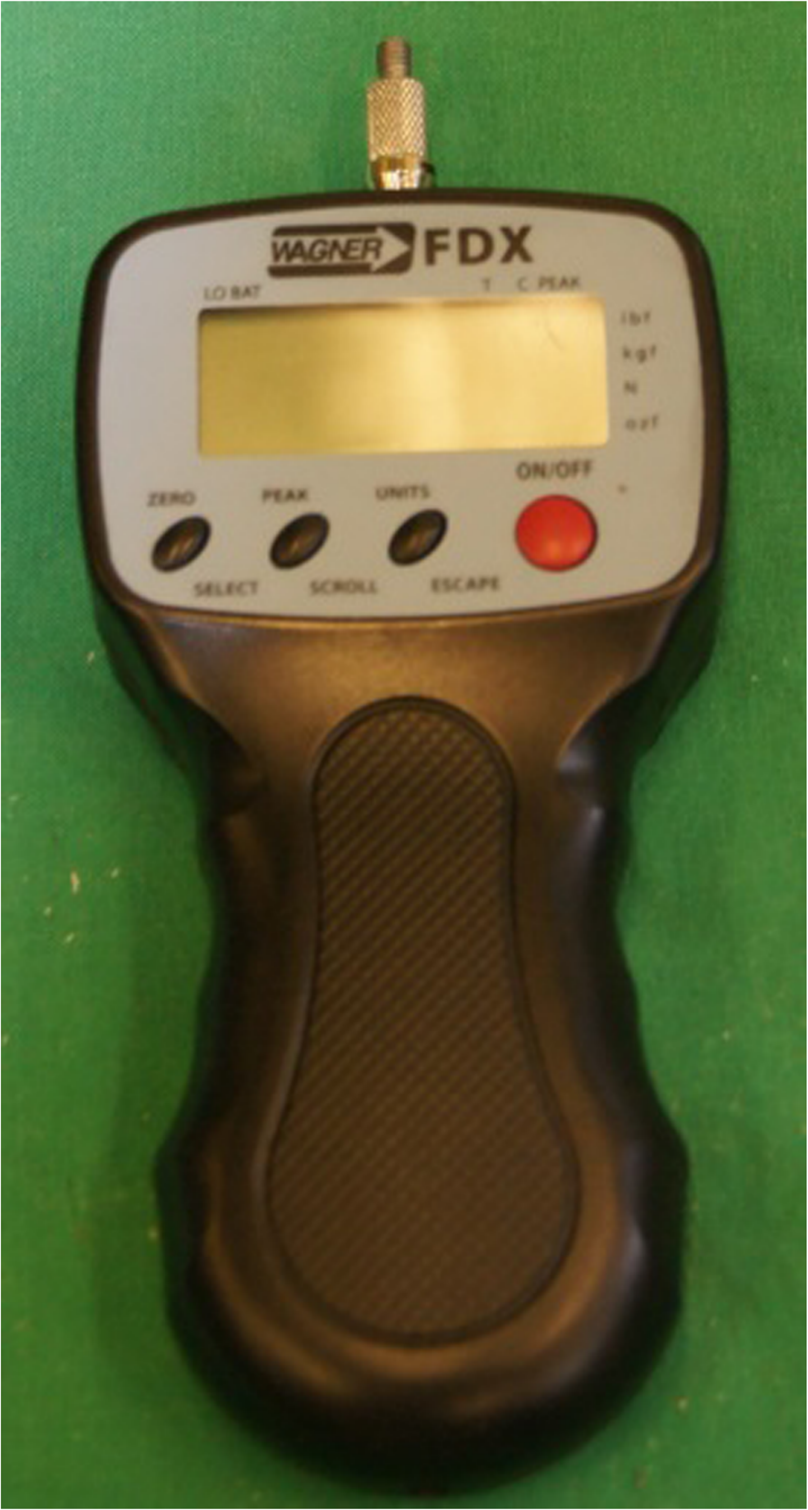
The Algometer used in this study (Pain Test FDX 100, Wagner Instruments Inc., Greenwich, Conn., USA)

**Fig. 2:**
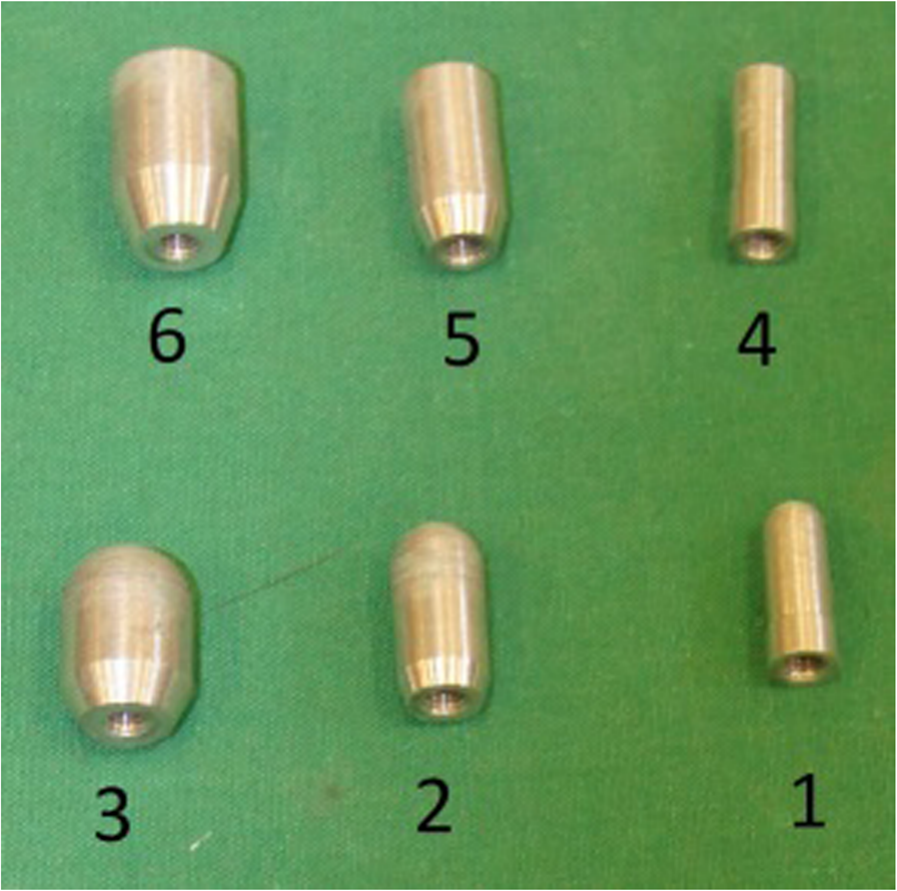
The algometer tips used in this study, tips 1–3 with semisperical and tips 4–6 with cylindrical shape

Measurements were carried out using a Pain Test FDX 100 Algometer (Wagner Instruments) (Fig. 1) and results were noted in Newton (N). Six aluminium tips were specifically made for this study (ADT Anker Datentechnik GesmbH) three hemispherical and three cylindrical tips with surface areas of 0.5cm^2^, 1cm^2^, and 2 cm^2^ (Fig. 2).

**Fig. 3:**
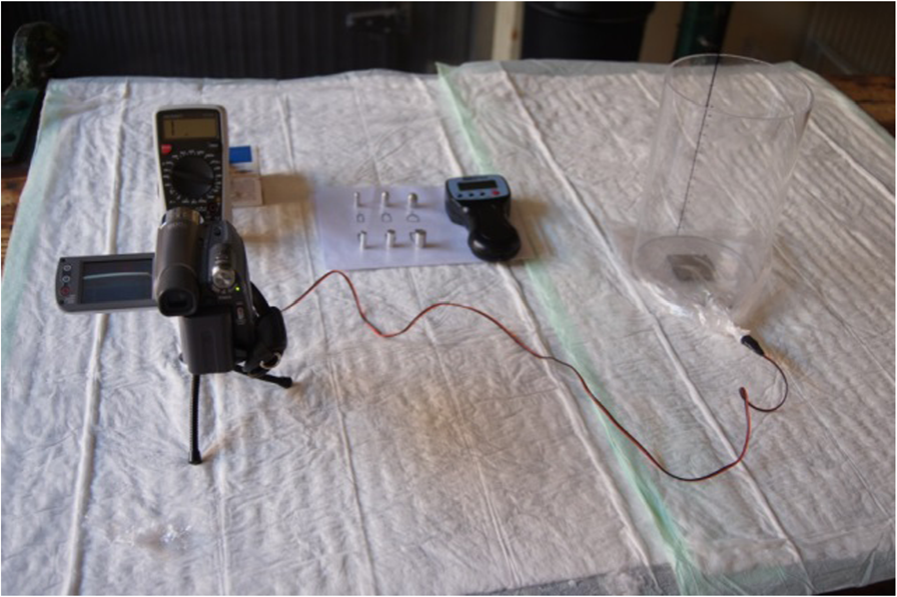
Measurement set-up for the post mortem specimens. On the *left* there is multimeter and the video camera, in the *middle* is the algometer with the tips used and on the *right* is the pressure sensor below the plexiglass cylinder later containing the tissue section

For the investigation of the post mortem specimens, a pressure sensor (FSR-152 NS) and a multimeter (Multikraft Multimeter VC 666) were used to translate the pressure exerted to the bottom of the into electrical resistance. In order to position the specimens in a standardized way, they were introduced into a Plexiglas cylinder (Fig. 3). The readings of the multimeter were documented using continuous video recording.

**Fig. 4:**
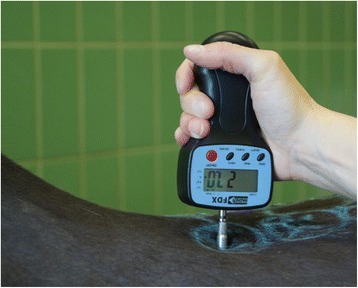
Algometry measurement at the level of the 14th thoracic vertebra (Pain Test FDX 100 Algometer)

All algometric measurements were carried out by one investigator (UP), and the pressure threshold was measured. This represents the nociceptive threshold in the live horses, and the pressure transfer threshold in the post mortem specimens. At the start of the in vivo measurements horses were accustomed to manipulations in their back area, and a back examination was carried out. The ensuing algometric measurements were carried out in such an order, that the algometer tips as well as the localizations of the thoracolumbar region were always changing, in order to avoid habituation effects as much as possible. A total of 216 measurements were carried out in each individual horse, with six localizations on each side of the back examined with each of the six algometer tips. The localizations examined were marked with a chalk about 5 cm paramedian at the level of T14, T16, and T18, as well as at the level of L1, L3, and L5, based on detailed palpation of the dorsal spinous processes during induced arching of the back The algometer was used in a perpendicular orientation (Fig. 4), and at first was held light contact with the skin for about 3 s, to reduce any reaction due to startle effects. Afterwards the pressure was gradually increased in 2 s–3 s intervals based on the studies of Haussler and Erb [4, 5] as well as Buthe and Hertsch [3] in order to create smooth transitions without abrupt pressure changes. As a positive reaction indicative of reaching nociceptive threshold behaviors such as turning of the ears, looking back at the examiner, stepping away from the examiner, hollowing the back away from the pressure as well as the sudden lifting of a limb with a stamping the foot to the ground or kicking. After the algometric measurements the examined localization were assessed ultrasonographically. This was done after the algometry measurements to avoid effects of habituation and/or skin irritation.

**Fig. 5:**
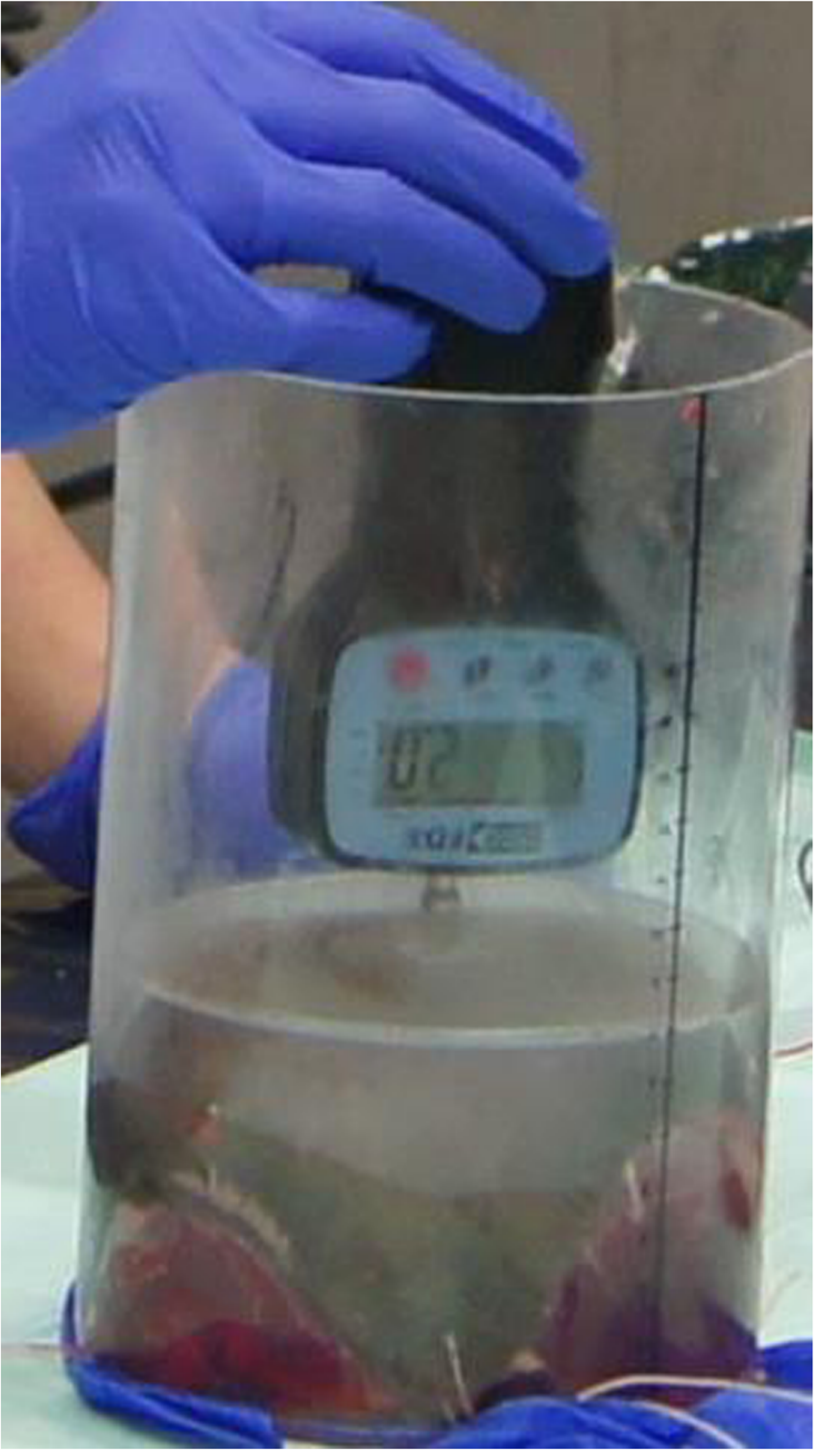
Pressure application with the algometer to the middle of a back tissue section

The postmortem specimens of the thoracolumbar region were prepared as follows: At the level of T14/T15, T18/L1, and L5/L6 transverse sections of all the tissues including the skin lateral to the dorsal spinous processes and dorsal to the ribs and the transverse processes respectively were carefully isolated. The resulting six sections with a diameter of about 20 cm were placed in the Plexiglas cylinder to avoid spreading of the tissues with the application of pressure. Prior to the measurement, the tissues were also assessed ultrasonographically. The different algometer tips were first applied at full tissue thickness, after removing the ventral half of the tissue at half the original tissue thickness and lastly with only the skin and the subcutaneous layers. The algometer was placed in the middle of the Plexiglas cylinder and pressure was exerted perpendicularly (Fig. 5). Results of the reduction in the electrical resistance of the pressure sensor placed below the tissue were noted at 5 N, 10 N, 15 N, 20 N, and 25 N.

In order to allow the calculation of the pressure sensor readings in ohm (Ω) of this setup into N, measurements of the algometer perpendicularly direct onto the pressure sensor were carried out.

### Statistical analyses

For statistical analyses, the software R (R Version 3.1.2 (2014–10-31)) was used. Levels of significance were set at *p* < 0.05. Medians and quartiles were calculated and presented. Mean values and standard errors were calculated.

## Results

There was no significant difference between body sides (F(df = 1;1941) = 0.11; *P* = 736), therefore the results were pooled.

### In vivo

#### Influence of the surface area and shape of the algometer tips

**Fig. 6:**
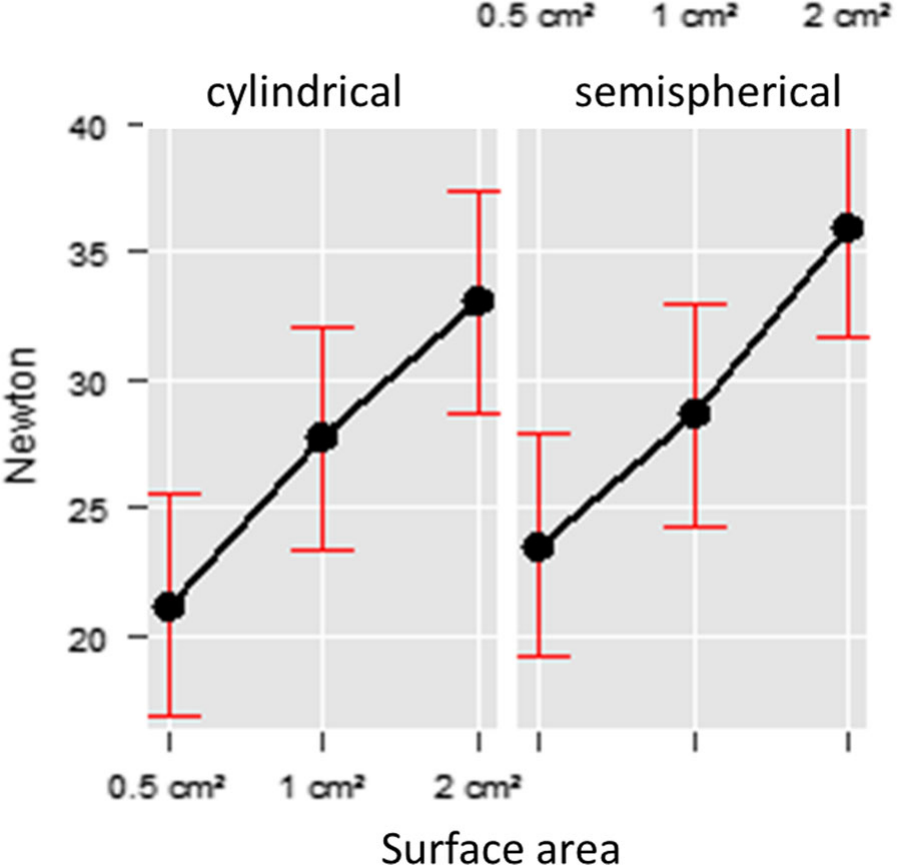
Linear regression of the pressure pain threshold of the thoracolumbar region of horses using 6 different algometer tips (cylindrical, hemispherical; surface area 0.5 cm2, 1 cm2, 2 cm2) The mean values and the confidence intervals are given

Both the surface area (0.5 cm2/1 cm2/2cm^2^; Chi^2^(df = 2) = 1147.08, *p* < 0.001) as well as the shape of the tips (hemispherical vs cylindrical; Chi^2^(df = 1) = 43.64, *p* < 0.001) showed an effect on the pressure threshold. The cylindrical tip with a surface of 0.5cm^2^ resulted in the lowest pressure threshold (median = 19.6 N [16.6, 23.9]), while the hemispherical tip with 2cm^2^ surface resulted in the highest pressure threshold (median = 34.0 N [30.0, 40.0]). The tips with a surface of 1cm^2^ lead to very similar results (hemispherical median 25.9 N [23.6, 31.7], cylindrical median = 25.1 N [23.0, 29.8]). The regression analysis showed the differences comparing the shape (hemispherical vs cylindrical) with a coefficient of B = 2.31 (SE = 0.35), while the regression analysis of the surface areas resulted in an increase of B = 6.51 (SE = 0.35) between 0.5cm^2^ and 1cm^2^, as well as B = 11.83 (SE = 0.35) between 1cm^2^ and 2cm^2^. Using the tips with the 2 cm2 surface, the widest distribution of results was obtained (Fig. 6).

#### The effect of anatomical location and tissue thickness

**Fig. 7:**
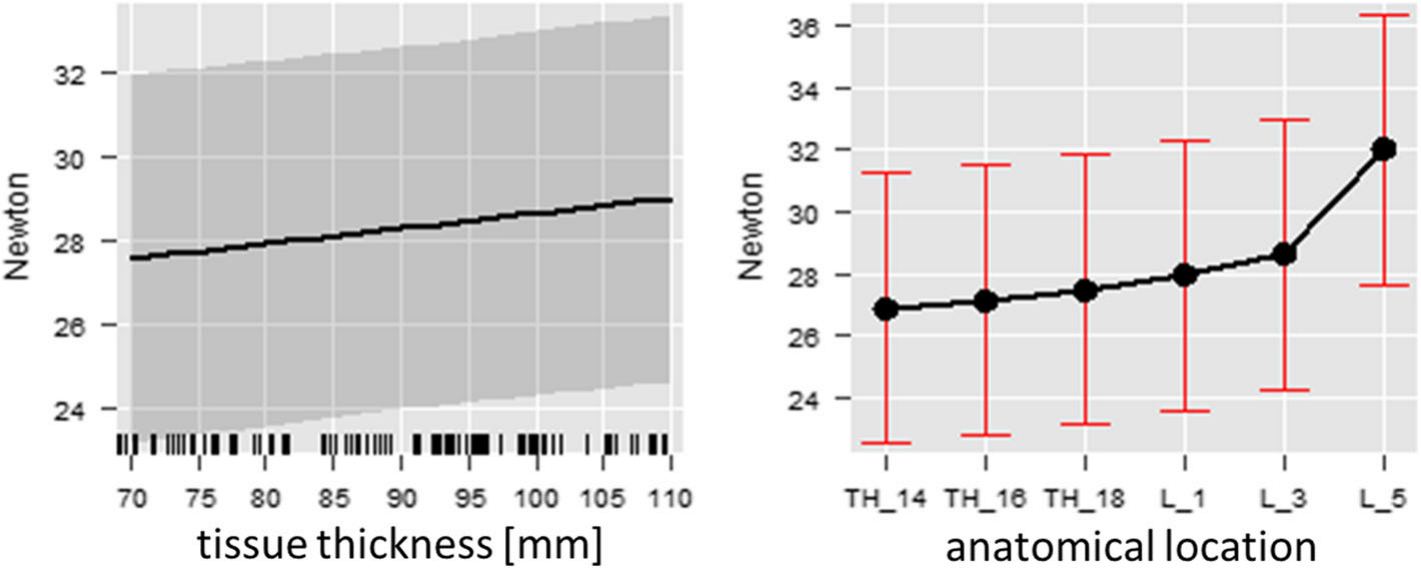
Linear regression of the tissue thickness (*left*) and the anatomical regions investigated (*right*). Mean values and confidence intervals are given. A clear parallel can be seen between the two

Tissue thickness had a noticeable effect on the pressure threshold as well as on the spread of the results. With increasing tissue thickness (measured sonographically) the pressure pain threshold increased (Chi^2^(df = 1) = 2.94, *p* = 0.086), as well as the variability of the results. A larger effect was found for the influence of the anatomical location (Chi^2^(df = 5) = 119.63, *p* < 0.001) with an increase of the pressure threshold at a tissue thickness of 7 cm (mean 27.59 N 95%Ci = [23.19, 31.99]) and at a tissue thickness of 11 cm (mean 29.04 N 95%Ci = [24.65, 33.42]). With a regression coefficient of B = 0.04 (SE = 0.02) pressure threshold increased with increasing tissue thickness, as is the case from cranial to caudal along the thoracolumbar region. The largest increase in pressure threshold was found between L3 and L5, with a regression coefficient of B = 5.08 (SE = 0.66) (Fig. 7).

#### In vitro

There was a linear relationship (Chi^2^(df = 1) = 27,428.25, *p* < 0.001) between the pressure exerted onto the pressure sensor and the resulting decrease in electrical resistance with a regression coefficient B = −0.12 (SE = 0.00). Pressure of 5 N directly onto the pressure sensor created 3.90 kΩ 95%Ci = [3.63, 4.18], 10 N 2.14 kΩ 95%Ci = [1.99, 2.29], 15 N 1.16 kΩ 95%Ci = [1.08, 1.25], 20 N 0.64 kΩ 95%Ci = [0.59, 0.68] and 25 N 0.35 kΩ 95%Ci = [0.32, 0.38]. Based on the manufacturer’s description of an inverted exponential function characteristic for the sensor the results in kΩ were transformed logarithmically.

#### The effect of tip surface area and tip shape

**Fig. 8:**
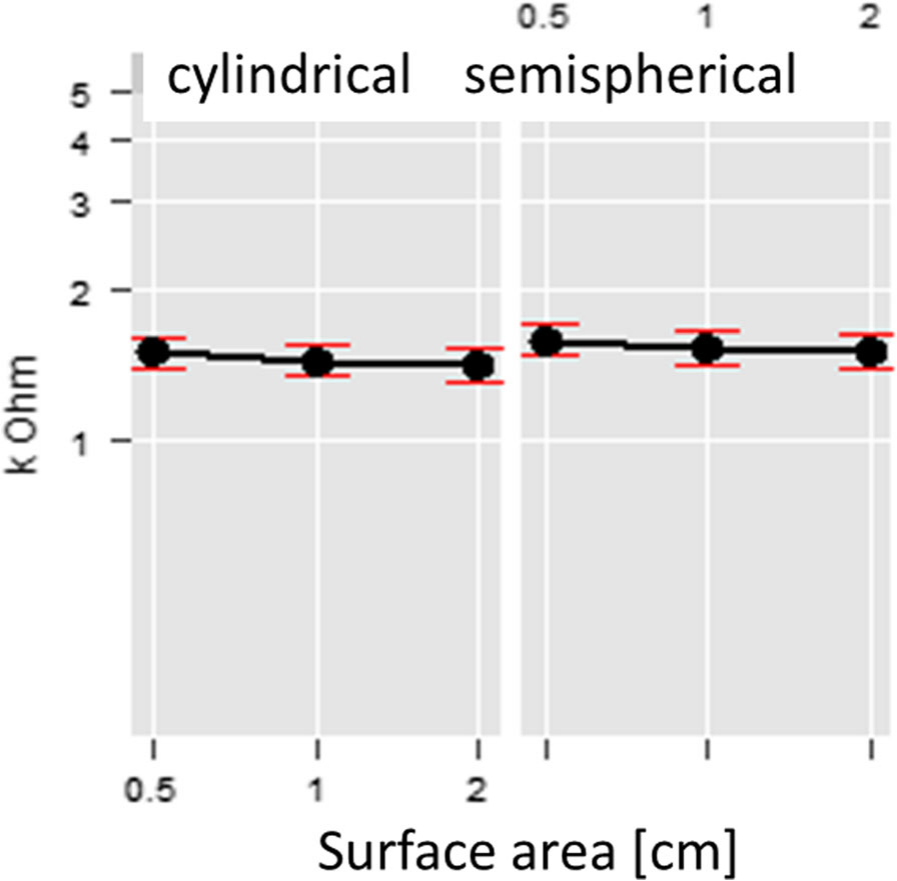
Linear regression of the effects of surface area and shape of the algometer tips on tissue pressure transmission

The first recording of pressure below the tissue specimen shows a marked influence of the size of the tip surface, with smaller surface areas transmitting pressure at much lower levels than larger surface areas. This effect is significantly independent of tissue thickness (Chi^2^(df = 2) = 1836.90, *p* < 0.001). The shape of the tip only created small differences (*p* = 0.015 (Chi^2^(df = 1) = 5.97)) in pressure transmission through the tissue (Fig. 8), with differences of 0.1 N–0.15 N between cylindrical and semispherical tips. During the measurements of the skin and subcutaneous tissues only the smallest variation of results was obtained, with results of median 0.6 N [0.5, 0.7] at 0.5 cm^2^, 0.9 N [0.7, 1.0] at 1 cm^2^ and 1.1 N [1.0, 1.2] at 2cm^2^. The difference between cylindrical tips (median 0.9 N [0.7, 1.1]) and hemispherical tips (median 0.8 N [0.6, 1.0]) was much smaller. With increasing pressure exerted on the tissues the effect of the surface areas decreased (between 0.5 cm^2^ and 1 cm^2^ B = −0.04 (0.02); between 1 cm^2^ and 2 cm^2^ B = −0.06 (0.02); Chi^2^(df = 2) = 8.31, *p* = 0.016), and the effect of the shape decreased as well, with a tendency of cylindrical tips transmitting more pressure than hemispherical tips (B = 0.06 (SE = 0.02); *p* = 0.003, Chi^2^(df = 1) = 8.81).

#### Effect of anatomical location and tissue thickness

**Fig. 9:**
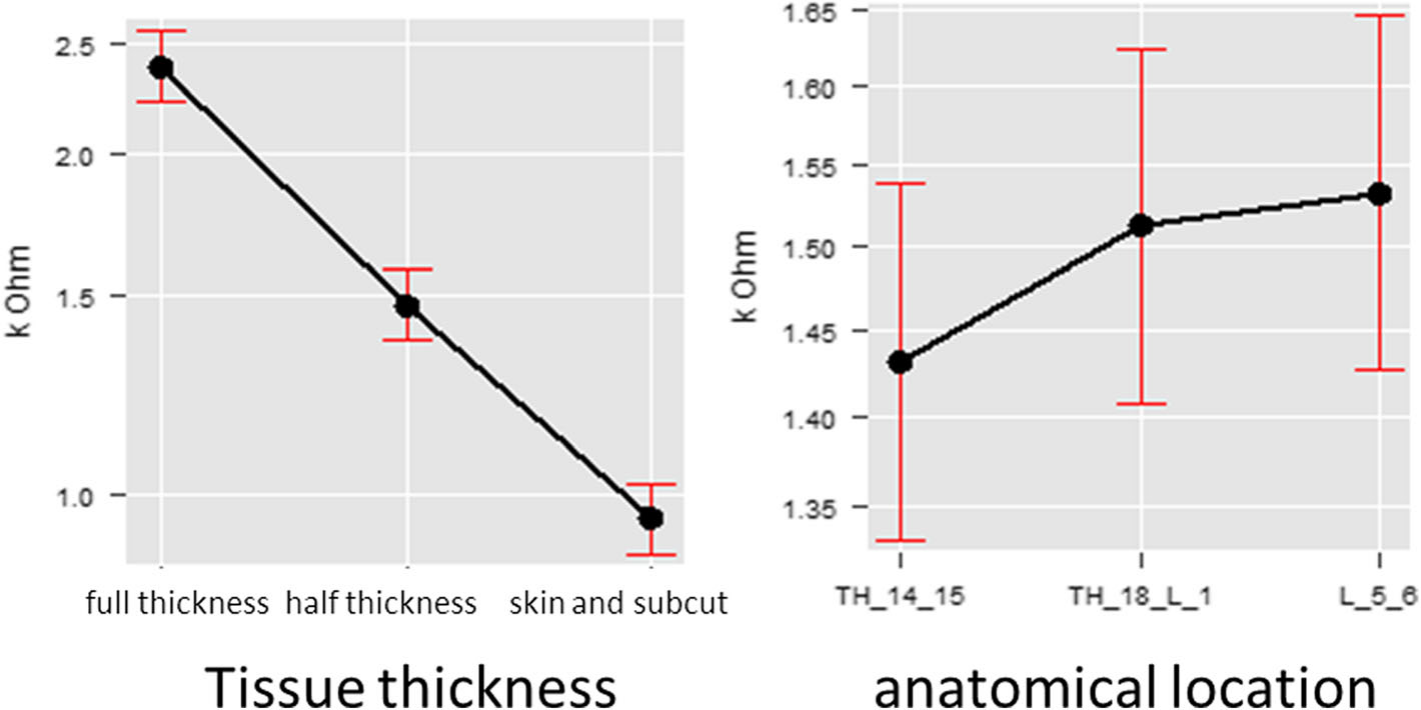
Linear regression of the effects of tissue thickness (*left*) and anatomical location (*right*) on the pressure transmission from an algometer through thoracolumbar tissues of horses. The reduction of kΩ values indicates an increase in pressure measured below the tissue

There was a linear relationship between the pressure transmission and the thickness of the tissue (Chi^2^(df = 2) = 4171.13, *p* < 0.001) with a regression coefficient of the effect of tissue thickness on the pressure transmission of B = −0.48 (SE = 0.01) between full and half thickness tissue samples, and B = −1.92 (SE = 0.01) between half thickness and skin/subcutaneous samples (Fig. 9). The marked effect of the anatomical location (Chi^2^(df = 2) = 25.46, *p* < 0.001) was again noted, with pressure transmission decreasing from cranial to caudal, both between T14/T15 and T18/L1 (B = 0.05; SE = 0.01) and between T18/L1 and L5/L6 (B = 0.07; SE = 0.01).

## Discussion

In the present study we have sought to carry out the algometry in a standardized fashion, based on the current literature [19]. However, we did not repeat the measurements even though this might have increased the value of our findings. The high number of measurements in each live horse (36 times 6) did not allow a full measurement repetition, but, as an indication of repeatability of results single measurements might have been repeated without creating undue stress to the horses. This should be seen as a limitation of the study.

Algometry is a non invasive method to quantify pain sensation in horses quantitatively and easily [9, 20]. However, algometry is not absolutely objective, as it depends on the pain reaction of the subject studied [12] and therefore the different reactivity individuals will create different results even though the pain experienced might be the same. The individuality of sensation as well as the individuality of reaction to sensation has been discussed repeatedly in humans [21], as well as in animals [12, 20, 22, 23]. Breed differences might be explained by a genetic selection for the coding of neurotransmitters and neuromodulators [24] or by a selection for placid demeanor. In the present study, the live horses were Warmbloods and Standardbred Trotters, and the results showed relatively small variations, indicating that these breeds might not be too different in their sensation as well as in their reactivity. The time of day also appears to influence the pain sensation of horses [22], and there is the possibility that horses might learn to avoid pressure by showing an earlier reaction. In the present study horses were measured during daylight hours over two consecutive days, but no standardized time was used, as this would not have been logistically possible.

It is not surprising that the results of the present study showed smaller algometer tips to lead to lower pressure thresholds than larger tips, similar to round surfaces being tolerated better than surfaces with an edge. Parallel results were found in the post mortem study, where the pressure transmission was similarly affected by surface area and shape. The results of present study using the 1 cm2 surface tips are comparable to other studies also using tips of this surface area [2, 4, 5, 9, 20, 22, 25, 26]. However, no other study used aluminium tips similar to the ones used in the present study, but all of them used a rubber surface, the mechanical qualities of which have not been reported. As the rubber is expected to have some elasticity, and this elasticity might potentially be variable in different temperatures, we elected to use the hard and stable material of aluminium for our investigation. Other algometry investigations have used tips of 2 mm to 14 mm in diameter [3, 10, 11, 23], and these results cannot be directly compared with the results of the present study, as the effect of surface area could be clearly shown. Smaller tips reduced the pressure threshold in the present study as well as in previously published studies [23, 27] and they also created the most consistent results.

In human algometry, surface areas of 0.5 cm^2^ or 1 cm^2^ have been used successfully for quantification of pain in the deeper layers of muscles [12, 15]. Small algometer tips (e.g. 0,2 mm diameter) deform the skin surface and activate intraepidermal nerve endings before relevant pressure is achieved in the deeper tissues, whereas surface areas of 1cm^2^ create a skin deformation as well as pressure in the deeper tissues, allowing also the evaluation of these deeper tissues [17]. For the measurement of muscular pain, algometer tips of more than 1.6 mm diameter are therefore recommended [16].

Tissue thickness and anatomical location are closely related as the tissues above the bony structures of the back (ribs in the thoracic region and transverse processes in the lumbar region) increase in thickness from cranial to caudal. This increase in tissue thickness is mainly due to an increase in thickness of the long back muscle as was shown sonographically [28]. In the present study the increase of the pressure threshold (i.e. the reduction of pressure transmission) from cranial to caudal was clearly documented in vivo as well as in vitro. The effect of an increase in the pressure threshold in the present study was most obvious in the lumbar region; this is similar to other studies [11, 20, 22, 29]. This marked increase in pressure threshold the lumbar region is not the consequence of a marked increase in tissue thickness at this level. For humans, where a similar increase was found, a variation in the density of neurovascular structures at this level is discussed [29, 30].

The tissue quality in addition to the tissue thickness is of relevance for the transmission of pressure exerted by an algometer. In the present study, all of the measurements in vivo were carried out over muscled areas of the back, while the pressure threshold over bony areas was found to be much smaller in a human study [14], as well as in a study on the pressure threshold in horses over the dorsal spinous processes [3]. This is reflected in the results of the in vitro part of the present study, where the skin and subcutaneous tissue samples showed direct, nearly unattenuated pressure transmission. In this context it is important though, that the reduction in muscle mass commonly present in horses with prolonged thoracolumbar pain and the reduction of the pressure pain threshold due to the reduction of muscle volume are not interpreted as two independent indications of thoracolumbar pain and dysfunction.

## Conclusions

Based on the results of this study, tissue thickness and tissue character as well as anatomical location should be considered for the interpretation of algometry results of the equine back, with cranial thoracolumbar areas, as well as areas with less tissue thickness, especially less muscle, creating lower pressure thresholds even in horses without any clinical signs of back pain. The algometer tip used should also be carefully chosen regarding its shape and surface area, with smaller tips creating lower pressure thresholds, and rounded tips creating higher pressure thresholds.

## Abbreviations

L: Lumbar vertebra
T: Thoracic vertebra
